# Emerging therapeutic agents in multiple myeloma: highlights from the 2024 ASH annual meeting

**DOI:** 10.1186/s40364-025-00803-0

**Published:** 2025-07-01

**Authors:** Qing Zhang, Yongping Song, Keshu Zhou, Quande Lin

**Affiliations:** 1 Department of Hematology, The Affiliated Cancer Hospital of Zhengzhou University and Henan Cancer Hospital, Zhengzhou, 450008 China; 2 Department of Hematology, The First Affiliated Hospital of Zhengzhou University, Zhengzhou, 450052 China

**Keywords:** Multiple myeloma, Tri-specific antibody, CELMoD, Cevostamab, Lisaftoclax, Inobrodib, Elranatamab, Mezigdomide, ISB 2001

## Abstract

Novel agents with innovative mechanisms of action were updated at the latest 2024 ASH Annual Meeting, some featuring first trials in combinations. Advances span new antibodies, bispecific T-cell engagers, next-generation CELMoDs, and agents targeting previously unexplored pathways in relapsed/refractory multiple myeloma. Key updates include: Cevostamab (FcRH5×CD3) demonstrated a 30.2% overall response rate in patients who underwent BCMA-targeted treatment and 60.6% in BCMA-targeted naïve patients;the triple-step dosing strategy reduced cytokine release syndrome. Lisaftoclax (APG-2575, BCL-2 inhibitor) displayed overall response rates ranging from 61.3 to 100% and ≥ VGPR rates of 32.3–100%, supporting enhanced response depth with favorable safety in combination regimens. Inobrodib (CCS1477, p300/CBP inhibitor) plus lenalidomide achieved a 71% overall response rate in pomalidomide-refractory patients, marking the first oral epigenetic modulator to reverse immunomodulatory drug resistance. Elranatamab (ELRA, BCMA×CD3) combined with carfilzomib and dexamethasone yielded an 83.3% overall response rate with a median duration of response not reached. Mezigdomide (MEZI, CELMoD) showed an 85.7% overall response rate and 17.5-month progression-free survival in Lenalidomide-refractory patients. ISB 2001 (BCMA×CD38×CD3 tri-specific antibody): achieved a 90% overall response rate at ≥ 50 µg/kg in heavily pretreated patients, with low-grade cytokine release syndrome observed. Through multi-targeted design, reversal of drug resistance mechanisms, and optimized combination strategies, treatment approaches for relapsed/refractory multiple myeloma are evolving toward greater precision, durability, and individualization.

To the editor,

Despite recent advancements in multiple myeloma treatment, some patients still experience inferior outcomes due to resistance mechanisms. We summarized the latest reports on several novel agents from the 2024 ASH Annual Meeting.

## Cevostamab

Cevostamab, a bispecific antibody (BsAb) targeting Fc receptor-homolog 5 (FcRH5) and CD3, has demonstrated significant efficacy in relapsed/refractory multiple myeloma (RRMM) [1]. An ongoing Phase I study updated efficacy and safety data from patients receiving 160 mg, showing an overall response rate (ORR) of 43.1% (72/167), including 6.6% achieving complete response (CR), 6.6% stringent CR (sCR),12.6% very good partial response (VGPR), and 17.4% partial response (PR). Prior BCMA-targeted patients showed a 30.2% ORR versus 60.6% in BCMA-targeted naïve patients. The reduced efficacy may be due to T-cell dysfunction and immune microenvironment exhaustion after BCMA-targeted therapy [2]. The most common treatment-emergent adverse events (TEAEs) were cytokine release syndrome (CRS) in 74.3%, which decreased to 63.3% with a triple-step dosing regimen; immune effector cell-associated neurotoxicity syndrome (ICANS) in 13.2%, and a grade ≥ 3 infection rate of 19.2%.All events were considered manageable [3].

## Lisaftoclax (APG-2575) combination

Lisaftoclax (APG-2575), a novel BCL-2 inhibitor, was evaluated in combination with pomalidomide (POM) and dexamethasone (DEX) (Arm A) or daratumumab (DARA), lenalidomide (LEN), and DEX (Arm B) for RRMM. In Arm A (*n* = 35), lisaftoclax was administered orally at escalating doses of 400–1200 mg. The ORR was 61.3% (19/31), with 9.7% achieving CR, 22.6% VGPR, and 29.0% PR. In Arm B, patients received 600 mg; among evaluable patients (*n* = 4), 50% of patients achieved CR, and the rest achieved VGPR or better. Grade ≥ 3 TEAEs included neutropenia (14.3%) and febrile neutropenia (2%) [4], the regimen enhanced response depth with no overlapping toxicity.

### Inobrodib (CCS1477) combination

Inobrodib (CCS1477), a first-in-class oral inhibitor targeting histone acetyltransferases P300 and CBP [5], had demonstrated promising efficacy in a Phase I/IIa trial involving 48 patients when combined with POM and DEX. In the highest dose cohort, ORR was achieved in 75% (6/8). Among POM-refractory patients, ORR was 71% (5/7), while 17% (2/12) of POM-naïve patients attained CR and minimal residual disease (MRD) negativity. The most frequently reported grade 3/4 TEAEs were hematologic toxicities (56%) and infections (29%), with no significant overlapping toxicity observed [6]. This represents the first fully oral epigenetic modulator regimen capable of reversing IMiD (immunomodulatory drugs) resistance.

### Elranatamab (ELRA) combination

ELRA, a subcutaneous CD3×BCMA BsAb, showed promising efficacy and durable responses with acceptable safety in the MagnetisMM-3 study [7]. In the Phase Ib MagnetisMM-20 study, 12 patients received ELRA combined with carfilzomib (CFZ) and dexamethasone (DEX), achieving an ORR of 83.3% with a median duration of response not reached. Grade 3/4 TEAEs included neutropenia (33.3%), thrombocytopenia (25%), anemia (25%), and lymphopenia (25%), and so on. No ICANS events were reported [8].

### Mezigdomide(MEZI) combination

MEZI is a novel, potent oral Cereblon E3 ligase modulator (CELMoD) that shows remarkable efficacy in IMiD-refractory patients [9]. In the Phase I/II CC-92,480-MM-002 trial, MEZI combined with DEX and BORT (MeziVd) or DEX and CFZ (MeziKd) demonstrated significant efficacy and manageable tolerability. In dose-escalation cohorts A and C, 28 and 27 Lenalidomide-refractory patients received MeziVd and MeziKd, respectively. The ORR was 75.0% (21/28) for MeziVd and 85.2% (23/27) for MeziKd, with ≥ VGPR rates of 39.3% and 44.4%. Median progression-free survival (PFS) was 11.8 months for MeziVd and 13.5 months for MeziKd. In the MeziVd dose-expansion cohort (D) involving 49 patients, the ORR was 85.7% (42/49), with 63.3% achieving ≥ VGPR, and a median PFS of 17.5 months [10]. This represents the longest reported in IMiD-refractory patients, while maintaining manageable safety.

## ISB 2001

ISB 2001 is a novel first-in-human BCMA×CD38×CD3 tri-specific antibody that overcomes antigen escape through multi-target synergy [11]. In the initial Phase I study, 14 patients were enrolled, including 9 with penta-exposure. The ORR was 75% (9/12) across all dose levels and reached 90% in patients receiving ≥ 50 µg/kg. Responses included sCR with MRD negativity (8.3%), VGPR (16.7%), and PR (50.0%). Most TEAEs were low-grade CRS, with no ICANS reported [12].

These new agents may provide promising options for multi-refractory patients. Future studies will focus on defining optimal sequencing and combinations to advance biomarker-guided precision therapy for RRMM.(Tables 1 and 2).

**Table 1:** Properties of novel agents in multiple myeloma

Agent	Target	Property	Mechanism of action	Administration	References
Cevostamab	FcRH5×CD3	IgG	BiTE	IV	[3]
Lisaftoclax (APG-2575)	BCL-2	Small molecule	Inhibitor	Oral	[4]
Inobrodib (CCS1477)	HATs(p300&CBP)	Small molecule	Inhibitor	Oral	[6]
Elranatamab(ELRA)	BCMA×CD3	IgG2κ	BiTE	SC	[8]
Mezigdomide(MEZI)	CELMoD	Small molecule	E3 ligase modulator	Oral	[10]
ISB 2001	BCMA×CD38×CD3	IgG1/ IgG3	Trispecific antibody	IV	[12]

BCMA, B-cell maturation antigen; BiTE, Bispecific T-cell Engager; CELMoD, Cereblon E3 ligase modulator; ELRA, Elranatamab; FcRH5, Fc receptor homolog 5; HATs, Histone acetyltransferases; IV, intravenous; MEZI, Mezigdomide; SC, subcutaneous

**Table 2:** Outcomes of clinical trials on novel agents and regimens in multiple myeloma

Agent		Cevostamab	Lisaftoclax (APG-2575) Combination		Inobrodib (CCS1477) Combination	Elranatamab(ELRA) Combination	Mezigdomide (MEZI) Combination			ISB 2001
Study		GO39775	APG2575MU101		NA	MagnetisMM-20	CC-92,480-MM-002 trial			NA
Phase		I	Ib/II		I/IIa	Ib	I/II			I
Regimen		Monotherapy	Lisaftoclax + POM + DEX	Lisaftoclax + DARA + LEN + DEX	Inobrodib + POM + DEX	ELRA + CFZ + DEX	MEZI + DEX + BORT(MeziVd) dose-escalation	MEZI + DEX + CFZ(MeziKd) dose-escalation	MEZI + DEX + BORT(MeziVd) dose-expansion	Monotherapy
Patients		167	35	7	48	12	28	27	49	14
mFU		11.3 m (range:0.5–42.8)	NA	NA	5.9 m (range:1.27–20.01)	3.24 m (range:1.51–13.47)	13.6 m	15.2 m	18.3 m	2.2 m (rang:1.0 -6.6)
ORR		43.1%	61.3%	NA	75% (highest dose cohort)	83.3%	75.0%	85.2%	85.7%	75%; 90%(≥50 µg/kg)
CR		13.2%	9.7%	50%	17% (pom-naïve patients)	NA	NA			8.3%
≥ VGPR		25.7%	32.3%	50%	NA	NA	39.3%	44.4%	63.3%	25.0%
Grade ≥ 3 TEAEs	Hematological	Neutropenia(28.2%), Anemia(28.0%)	Neutropenia (14.3%), Febrile Neutropenia (2%)		Hematological(56%), Neutropenia(35%), Thrombocytopenia (27%), Anemia (13%);	Neutropenia(33.3%), Thrombocytopenia (25.0%), Anemia (25.0%); Lymphopenia (25.0%), Leukopenia(16.7%)	Neutropenia (35.7%), Thrombocytopenia (21.4%),	Neutropenia (44.4%)	Neutropenia (63.3%), Thrombocytopenia (26.5%)	NA
	Non-Hematological	CRS (1.8%), Cough(0.6%), Nausea(0.6%), Fatigue(1.2%), ICANS(1.8%), Infection (19.2%), HLH(1.2%); DIC with pseudomonal sepsis (0.6%)	NA		Infection (29%)	Fatigue (8.3%), Peripheral edema (16.7%), Increased blood alkaline phosphatase (16.7%), Pulmonary embolism (16.7%)	Infections (17.9%)	Infections (33.3%)	Infections (32.7%),	Urinary tract infection(7.1%)
Clinical trial number		NCT03275103	NCT04942067		NCT04068597	NCT05675449	NCT03989414			NCT05862012
References		[3]	[4]		[6]	[8]	[10]			[12]

BCMA, B-cell maturation antigen; BORT, Bortezomib; CELMoD, Cereblon E3 ligase modulator; CRS, Cytokine release syndrome; CAR-T, Chimeric antigen receptor T cells; CFZ, Carfilzomib; CR, Complete response; DEX, Dexamethasone; DARA, Daratumumab; DIC, Disseminated intravascular coagulation; ELRA, Elranatamab; HLH, Hemophagocytic lymphohistiocytosis; ICANS, Immune effector cell-associated neurotoxicity syndrome; LEN, Lenalidomide; m, months; MEZI, Mezigdomide; mFU, Median follow-up; NA, Not Applicable; ORR, Overall response rate; Patients, Patients; PR, Partial response; POM, Pomalidomide; TEAEs, Treatment-emergent adverse events; VGPR, Very good partial response

## Data Availability

No datasets were generated or analysed during the current study.

## Abbreviations

BCMA：: B-cell maturation antigen
BsAb: Bispecific antibody
BORT: Bortezomib
CELMoD: Cereblon E3 ligase modulator
CR: Complete response
CRS: Cytokine release syndrome
CAR-T: Chimeric antigen receptor T
CFZ: Carfilzomib
DEX: Dexamethasone
DARA: Daratumumab
ELRA: Elranatamab
FcRH5: Fc receptor homolog 5
ICANS: Immune effector cell-associated neurotoxicity syndrome
IMiDs: Immunomodulatory drugs
LEN: Lenalidomide
MEZI: Mezigdomide
MM: Multiple myeloma
ORR: Overall response rate
PR: Partial response
PFS: Progression-free survival
POM: Pomalidomide
RRMM: Relapsed/refractory multiple myeloma
sCR: stringent CR
TD: Target dose
TEAEs: Treatment-emergent adverse events
VGPR: Very good partial response
